# Effectiveness of acupuncture in the treatment of cyclic mastalgia: a study protocol for a randomized controlled trial

**DOI:** 10.1186/s12906-022-03779-8

**Published:** 2022-11-18

**Authors:** Chuan Yu, Jun Wang, Bin Shen, Xiang Li, Rui Zhang, Yan Qin, Guofan Jian, Jing Guo

**Affiliations:** 1 Department of Acupuncture and Moxibustion, Beijing Hospital of Traditional Chinese Medicine, Capital Medical University, No. 23 Meishuguanhou Street, Dongcheng District, Beijing, 100010 China; 2 Department of Acupuncture and Moxibustion, Beijng Traditional Chinese Medicine Hospital Pinggu Hospital, No. 6, Pingxiang Road, Pinggu District, Beijing, 101200 China

**Keywords:** Cyclic mastalgia, Acupuncture, Trial, Protocol

## Abstract

**Background:**

About 68% of women aged 18–44 years have experienced cyclic mastalgia (CM), which occurs during the luteal phase of the menstrual cycle when elevated hormone levels induce greater breast gland thickness. CM has a moderate-to-severe impact on a woman’s quality of life. Prior research has suggested that acupuncture may be beneficial for breast pain relief. In this study, we investigate the effectiveness of manual acupuncture (MA) in the treatment of CM compared with that of sham acupuncture (SA).

**Methods:**

This is a multicenter, randomized, controlled trial. A total of 108 eligible CM patients will be randomly assigned to either MA (*n* = 54) or SA (*n* = 54) group using a 1:1 ratio and a stratified, blocked randomization. Acupuncture will be performed two weeks prior to menstruation and discontinued when menses begins. In both the MA and SA group, participants will be given acupuncture three times per week for 2 weeks per menstrual cycle for three consecutive menstrual cycles, encompassing a total of 18 sessions. The primary outcome will be the change in the average daily Breast Pain Visual Analog Scale (VAS-BP) over the first two weeks of menstruation from baseline to endpoints. The number of nominal days of breast pain (NDBP) two weeks before menstruation, World Health Organization Quality-of-Life Scale-Short Form scores, global patient assessment, breast glandular-section thickness, and breast-duct width three days before menstruation will also be measured as secondary outcomes.

**Discussion:**

This prospective randomized trial will help evaluate the efficacy of acupuncture in treating CM. The results of this study will provide evidence of the therapeutic effectiveness of acupuncture on CM.

**Trial registration:**

ClinicalTrials.gov Identifier: NCT05408377, registered on June 7, 2022.

## Administrative information

Note: The numbers in brackets in this protocol refer to SPIRIT checklist item numbers. The order of the items has been modified to group similar items together (see http://www.equator-network.org/reporting-guidelines/spirit-2013-statement-defining-standard-protocol-items-for-clinical-trials/).

## Background

Approximately 60 to 70% of women have been plagued by mastalgia during their lifetime [1], which accounts for up to 66% of physician visits for breast symptoms, and contributes to serious anxiety [2]. According to menstrual cycle, mastalgia can be divided into two types: cyclic mastalgia (CM) and noncyclic mastalgia. CM is more likely to occur in pre-menopausal women than in postmenopausal women. About 68% of women aged 18–44 years have complained about cyclic mastalgia [3].

Increased water retention in the breast and ductal ectasia has been suggested to be the principal causes of CM [4]. Breast volume may increase by more than 100 mL during the luteal phase of the menstrual cycle due to the hormonal stimulation of breast parenchyma [5, 6]. In addition, breast pain was found positively associated with the degree of ductal dilatation [7]. CM pain intensity in most patients may be similar to that caused by chronic cancer [8]. Women with frequent mastalgia often complain more severe pain, which seriously interfered with their daily lives [9]. However, there is still a lack of specific therapeutic guidelines for mastalgia. The first-line management of breast pain is proposed to be appropriate explanation, reassurance, and good bra-fitting advice [10]. Topical nonsteroidal anti-inflammatory drugs (NSAIDs) and endocrine drugs are effective for mastalgia relief [11], while accompanied by potentially serious adverse effects [4]. A majority of patients, however, respond favorably to nonpharmacologic measures [12], such as alternative treatment strategies like acupuncture.

Acupuncture analgesia has been widely used for patients with breast abnormalities for millennia in China. A network meta-analysis [13] has suggested the effect of acupuncture on relieving pain caused by mammary gland hyperplasia (MGH). However, the evidence cited in the meta-analysis is insufficient. It has been found that acupuncture can regulate estrogen secretion levels and restore hyperplastic mammary glands [14]. Additionally, acupuncture can effectively increase blood flow in lesion areas and reduce the resistance index of blood flow [15]. Previous studies showed that acupuncture may also be beneficial in relieving breast pain [16, 17]. In two studies, effectiveness comparisons were made between manual (MA) and sham acupuncture (SA), but the conclusions diverged [17, 18]. High-quality evidence of the effectiveness of acupuncture on CM is still lacking [10]. Hence we designed the multi-center, randomized, controlled trial with long-term follow-up to observe the analgesia effectiveness of acupuncture in the therapy of CM.

## Methods

### Objectives/hypotheses

The aim of this study is to evaluate the effectiveness of acupuncture in the treatment of CM. We have two hypotheses: (1) acupuncture can reduce breast pain, (2) acupuncture can decrease the thickened mammary glands and expanded mammary ducts.

### Trial design

**Fig. 1:**
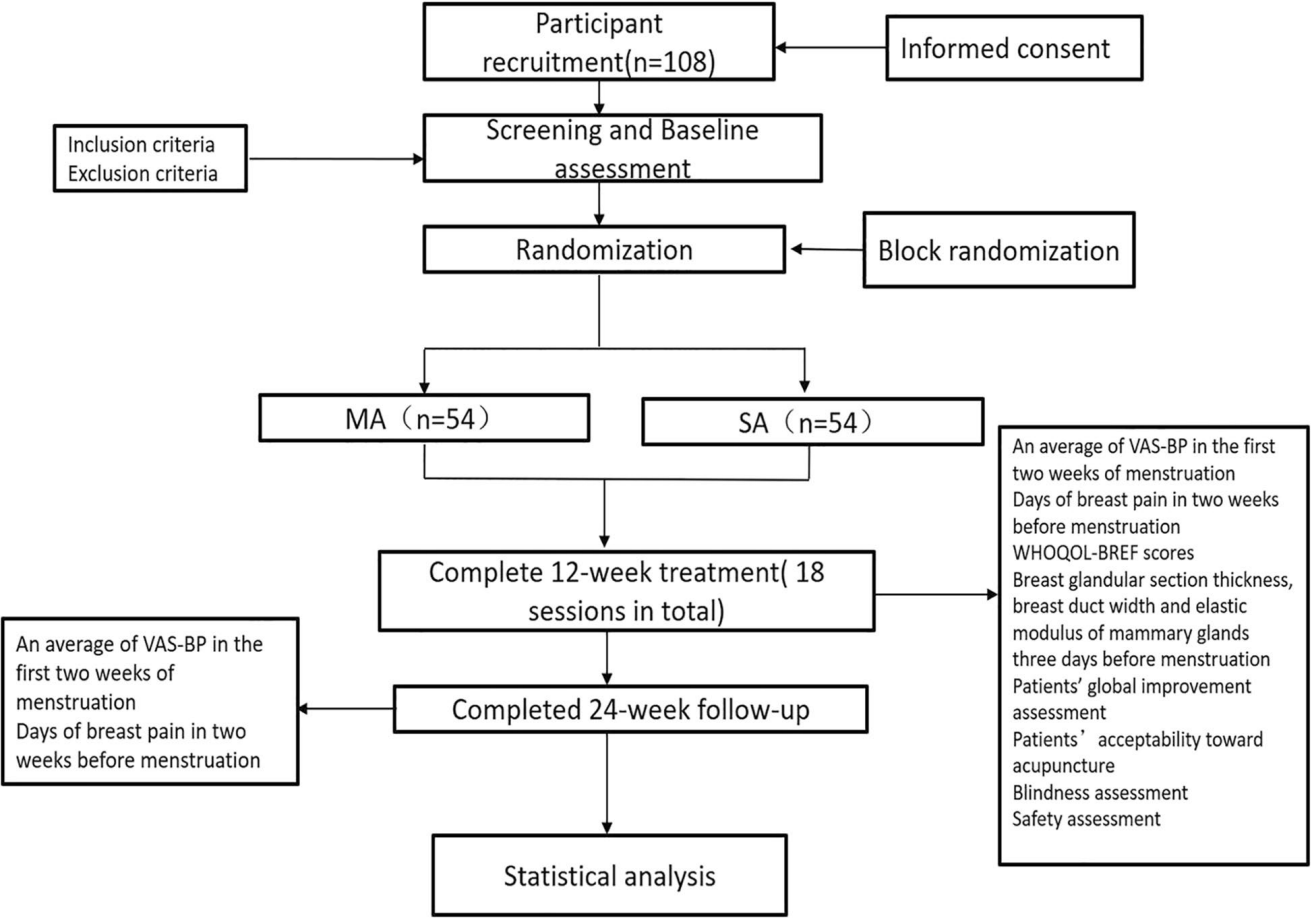
Flowchart of participants throughout the proposed trial

This study is a prospective randomized, patient-and-assessor-blinded, parallel-group trial. We applied the SPIRIT checklist [19] and the Declaration of Helsinki [20] to create our protocol, and the trial was approved by the Ethics Committee of the Beijing Traditional Chinese Medicine Hospital Pinggu Hospital (number 2022-sfzx-01) and registered at ClinicalTrials.gov (Identifier: NCT05408377). The flowchart of the trial is depicted in Fig. 1.

### Study setting

The research will be carried out in three Beijing-area hospitals: (1) Beijing Traditional Chinese Medicine Hospital Pinggu Hospital, (2) Pinggu Huangsongyu Community Hospital, and (3) Pinggu Xiagezhuang Community hospital. A total of 108 patients will be randomly assigned to MA and SA groups in a 1:1 allocation ratio.

### Eligibility criteria

#### Inclusion criteria

Patients who fulfill all of the following criteria will be included.

1. 18–45 years of age;
2. breast pain starting within two weeks before menses and improving after onset; pain that is dull, heavy, or aching, bilateral, poorly localized, and extends into the axilla [21, 22];
3. fulfillment of all conditions mentioned below: a. moderate or severe breast pain as indicated by a score of 3 or more on a scale of 1–10 on the VAS; b. history of CM for at least the past three consecutive cycles; c. ≥ 3 days and ≤ 14 days of premenstrual breast pain; and d. a history of regular menstrual cycles of 28 ± 3 days;
4. breast X-ray or breast ultrasonographic examination that excludes malignant breast lesions; and
5. signed informed consent and voluntary participation by the patient for the present study.

#### Exclusion criteria

Patients who fulfill any of the following criteria will be excluded.

1. those who accepted acupuncture or drugs to treat breast pain within one month prior to entering the study;
2. those with breast inflammation, breast fibroma, breast cystic hyperplasia, or other benign breast lesions;
3. those with severe primary diseases of the cardiovascular system, pulmonary system, liver, kidney, or hematopoietic system;
4. those with a history of breast cancer among first-degree relatives;
5. those with breast pain caused by costochondritis, chest wall injury, rib fracture, or other extramammary pain diseases;
6. pregnancy or lactation;
7. those with serious mental illness;
8. poor patient compliance;
9. participation in other clinical trials within the last month;
10. patients with serious skin disease or infection at the acupuncture site.

The practitioner of the acupuncture treatment and ultrasonographic scans will meet the following criteria:

1. all acupuncturists will have a license and at least five years of acupuncture experience, and
2. all ultrasound physicians will have a license and at least five years of professional experience.

### Who will take informed consent?

All potentially eligible women with CM will be recruited and be required to give their phone numbers so that the researchers can contact them. Eligible participants will be explained the research in detail, including the potential benefits and possible risks of this study, and required to sign their informed consent.

### Interventions

#### Explanation for the choice of comparators

In this trial, all participants will be required to undergo acupuncture (MA) or SA two weeks before menstruation. To achieve the effect of blinding in patients, the manipulation of needles will be similar and acupuncture ritual is the same in both the MA and SA groups. This reflects the advantages of a favorable masking effect and a higher feasibility for the operation.

### Description of intervention

#### MA group

According to theory of TCM, its main acupoints are the Ashi point, Tanzhong (CV17), bilateral Wuyi (ST15), Rugen (ST18), Tianzong (SI11), Geshu (BL17), Ganshu (BL18), Hegu (LI4), Sanyinjiao (SP6), and Taichong (LR3). The location of the above acupoints is based on the World Health Organization Standard Acupuncture Locations, World Health Organization Western Pacific Region [23]. Sterile, stainless-steel needles are 0.3 mm × 40 mm in size (Hwato brand, Suzhou Medical Appliance Factory, China).

After local skin disinfection using 75% alcohol wipes and with the patients in a sitting position, the acupuncturists will fix an opaque pedestal over the acupuncture point. There is an adhesive pad below the pedestal to ensure that the entire pedestal adheres to the acupoints. Then, the acupuncturists will insert the needles into the acupoints through the adhesive pad. The depth of needling will vary based upon the participant’s body size. The location and insertion depth of these points are described in Table 1. After insertion, all needles will be manually manipulated (with equal manipulations of twirling, lifting, and thrusting) to achieve the deqi sensation—defined as a composite of unique needling sensations that include soreness, numbness, distention, or heaviness felt by both participants and acupuncturists [24]. All needles will be retained in position for 30 min and then gently removed.

**Table 1:** Acupoints used in the MA group

Acupoints	Locations	Needle insertion
Ashi point	The most obvious local pain point over the breast	Oblique needle insertion at depth of 15–20 mm
Tanzhong (CV17)	On the anterior midline, on the level of the 4th intercostal space, at the midpoint of the line joining the two nipples	Horizontally needle insertion at depth of 5–10 mm
Wuyi (ST15)	In the 2nd intercostal space, 4 cun lateral to the anterior midline	Horizontally needle insertion at depth of 15–20 mm
Rugen (ST18)	In the 5th intercostal space, vertically below the nipple	Horizontally needle insertion at depth of 15–20 mm
Tianzong (SI11)	In the middle of the infrascapular fossa	Perpendicular needle insertion at depth of 15–25 mm
Geshu (BL17)	On the back, under the spinous process of the 7th thoracic vertebra, 3 cun lateral to the posterior midline	Oblique needle insertion at depth of 15–20 mm
Ganshu (BL18)	On the back, under the spinous process of the 9th thoracic vertebra, 3 cun lateral to the posterior midline	Oblique needle insertion at depth of 15–20 mm
Hegu (LI4)	On the back of the hand, between the 1st and 2nd metacarpal bones, at the midpoint of the radial side of the 2nd metacarpal bone	Perpendicular needle insertion at depth of 15–25 mm
Sanyinjiao (SP6)	On the anterolateral side of the calf, 3 cun below the nose, a transverse finger (middle finger) from the front edge of the tibia	Perpendicular needle insertion at depth of 15–25 mm
Taichong (LR3)	In the depression anterior to the junction of 1st and 2nd metatarsal bones	Perpendicular needle insertion at depth of 15–25 mm

#### SA group

Non-penetrating sham acupuncture will be applied at 14 non-acupuncture points on the back. These points are located in different ganglionic segments from the breast pain area, which avoid segmental effects. Participants will experience superficial touch at sham points 1–14. The patients will be required to keep in a prone position. After local skin disinfection using 75% alcohol wipes, the pedestal will be affixed to non-acupuncture points, blunt tip needles of 0.30 × 40-mm size (Hwato brand, Suzhou Medical Appliance Factory, China) will be inserted into the pedestal at a depth of 3–5 mm until the needles remain erect. The needles will be allowed to touch the skin but will not be inserted into the skin, and each point will be lifted and twisted evenly three times. The patients will feel a pricking sensation similar to that of deqi, by simulating a puncture of the skin. All needles will remain in situ for 30 min and then be gently removed. The location of these non-acupuncture points are shown in Table 2.

**Table 2:** Locations of non-acupuncture points in the SA group

Non-acupoints	Locations
Sham point 1–2	Bilateral, at a distance of 20 mm horizontally outside from the spinous process of the 9th thoracic vertebra
Sham point 3–4	Bilateral, at a distance of 20 mm horizontally outside from the spinous process of the 10th thoracic vertebra
Sham point 5–6	Bilateral, at a distance of 20 mm horizontally outside from the spinous process of the 11th thoracic vertebra
Sham point 7–8	Bilateral, at a distance of 20 mm horizontally outside from the spinous process of the 1st lumbar vertebra
Sham point 9–10	Bilateral, at a distance of 20 mm horizontally outside from the spinous process of the 2nd lumbar vertebra
Sham point 11–12	Bilateral, at a distance of 10 mm horizontally outside from the 6 cun below the gluteal sulcus
Sham point 13–14	Bilateral, at a distance of 10 mm horizontally outside from the depression below the belly of the gastrocnemius m. when stretching the leg or lifting the heel

All patients will undergo therapy two weeks prior to menstruation, three times per week and six times per menstrual cycle for three consecutive menstrual cycles, with treatment ending when menstruation begins, encompassing a total of 18 sessions. Acupuncture treatment will be performed by licensed acupuncturists with at least 5 years of experience in practicing acupuncture. Patients will be treated individually behind a curtain. Both groups of patients will receive explanation and good bra-fitting advice. Patients will not be allowed to consume any other specialized medication or undergo any therapy for CM during the trial, and any additional details will be reported on the case report form (CRF).

#### Criteria for discontinuing or modifying allocated interventions

The following criteria used to terminate a study intervention: (1) when the participant voluntarily withdraws from the study, (2) when there are changes to their health status that make continued participation inadvisable, and (3) the presence of severe adverse events such as severe complications, hemorrhage, or unbearable acupuncture pain.

#### Strategies to improve adherence to interventions

At the beginning of the trial, we will inform all participants with respect to the entire protocol comprising this trial and will provide convenient treatment and evaluation times for the participants. We plan to set up a chat group using WeChat (WeChat, Version: 8.0.21, Tencent, Shenzhen, China) to maintain close contact with the participants and to improve adherence to the treatments.

#### Relevant concomitant care permitted or prohibited during the trial

Patients will not be allowed to undergo any other additional complementary treatments during the study protocol. If patients manifest intolerable breast pain during treatment, they will be treated with topical nonsteroidal anti-inflammatory drugs (NSAIDs) such as diclofenac. The types of medications and dosages used will be recorded on the CRF.

#### Provisions for post-trial care

If severe adverse effects due to acupuncture occur, the therapy will be halted immediately and the participant will be provided suitable follow-up treatment free of charge until the patient recovers.

### Outcomes

#### Primary outcome

The primary outcome is the change in the average of VAS-BP over the first two weeks of menstruation from baseline (two weeks before randomization) to the endpoints, as reported in a daily pain diary.

The VAS-BP will be measured using a 10-cm linear VAS with 0 representing no pain and 10 the worst imaginable pain. The average of VAS-BP will be calculated by dividing the sum of VAS-BP in the first two weeks of menstruation by the number of days of pain.

#### Secondary outcomes

The secondary outcomes are as follows:

1. The changes in the number of nominal days of breast pain (NDBP) during the two weeks prior to menstruation relative to baseline to endpoints.
2. The changes in WHOQOL-BREF scores from baseline at week 12. The WHOQOL-BREF China version will be used to evaluate the quality of life (QOL) of the participants. The WHOQOL-BREF Questionnaire consists of 26 items, in which the first and second questions of the questionnaire regard patient QOL and their health status in general. The next 24 questions assess QOL in four dimensions: physical health (seven items); psychological health (six items); environment (eight items); and social relationships (three items). Each item is answered on a five-point scale, with a higher score denoting a better QOL [25, 26].
3. The changes in breast glandular-section thickness and breast-duct width three days before menstruation from baseline at week 12.

Mammary ultrasonography will be used to observe breast glandular-section thickness and breast-duct width. All ultrasonographic imaging acquisition work will be completed by the same ultrasound physician with at least five years of professional practice.

4. Evaluation of the patient's global improvement. This will be assessed by a self-reporting pain level scale ranging from 1 to 7: significantly reduced, moderately reduced, slightly reduced, no change, slightly aggravated, moderately aggravated, and significantly aggravated, respectively. The proportions of patients reporting “significantly reduced” or “moderately reduced” will be recorded as the response rate for overall efficacy.
5. Patient acceptability of acupuncture at the end of the 1^st^ and 18^th^ treatments. The acceptability of acupuncture will be assessed among participants in the MA group using a 3-point index: unacceptable (0 points), acceptable (1 point), and easily accepted (2 points). Those women who regard acupuncture as unacceptable will report their reasons, and the average score will be calculated after the two assessments.
6. Blindness assessment. Within three minutes after the last treatment session in week 12, all participants will be asked to answer the question “Do you think you have received acupuncture treatment?”, and choose between the options “Yes” and “No.”

#### Safety assessment

All adverse events will be recorded on the CRF throughout the trial. Acupuncture-related adverse effects such as extreme pain, local hematoma, infection or abscess, or broken needles—as well as discomfort after treatment—will be recorded in detail in a timely fashion. Patients and evaluators will compile a full description of the categories and severity of the adverse events and any correlation with treatment. If the adverse event is severe and associated with the trial, the patient will be withdrawn from the study and afforded appropriate medical care.

#### Participant timeline

The study period is presented in Table 3. The duration of the trial will be 38 weeks for each patient, including a two-week baseline assessment (weeks 0–2), 12-week treatment period (weeks 1–12), and 24-week follow-up (weeks 13–36).

**Table 3:**
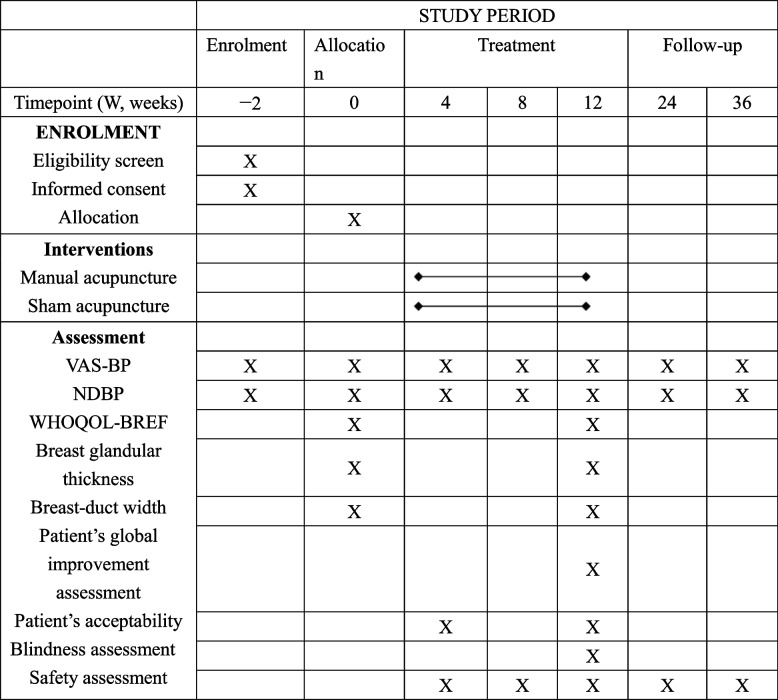
Study period

#### Sample size

Sample size estimation was based on changes in the breast pain VAS (VAS-BP) score. According to pre-trial results, the VAS-BP score significantly decreased by 2.98 ± 0.95 points in the MA group compared to 2.35 ± 0.97 in the SM group after treatment with respect to baseline. For the current trial, we assumed a significance level of α = 0.05 and a power (1-β) of 0.90. For a two-sided outcome, at least 42 participants will be required for each group as calculated using PASS version 11.0 (NCSS, LLC, Kaysville, UT, USA). We assumed a two-tailed test with a 20% drop-out rate, and thus a total of 108 patients (54 in each group) will be recruited.

#### Recruitment

Patients will be recruited via outpatient clinic, advertisements on websites, posters from hospitals, and friends’ circles using WeChat (WeChat, Version: 8.0.21, Tencent, Shenzhen, China). In addition, we will cooperate with the Gynecology and Physical Examination Departments of the hospital for potential patients to ensure the recruitment. All potentially eligible women with CM will be recruited and contacted by the researchers by phone to arrange an interview at hospital. Patients who meet the inclusion criteria will be invited to participate in the trial and informed about the potential benefits and possible risks of the study. All participants will be required to sign informed-consent agreements. After completing the baseline assessment, patients will be randomly assigned to either the MA or the SA group.

### Assignment of interventions: allocation

#### Sequence generation

Randomization will be performed by the Clinical Epidemiology Research Center of the Third Hospital of Peking University. Eligible patients will be randomized into the MA or SA group in a 1:1 ratio with stratified blocked randomization. Randomized sequence numbers will be produced by R Software 4.0.3 rm packages (Lucent Technologies, Murray Hill, NJ, USA).

#### Concealment mechanism

Randomization will be performed by an independent data manager who is not involved in the trial. Randomized sequence numbers will be maintained in sealed opaque envelopes, and the envelopes will be numbered.

#### Implementation

When a patient receives treatment for the first time, the envelope with its number corresponding to the sequence of assignment to the study assessment will be opened by the acupuncturist, and the group digit contained in the envelope will represent the treatment that the patient will receive.

#### Assignment of interventions: Blinding

Acupuncturists in this experiment cannot be blinded due to the unique nature of acupuncture manipulation. However, the group allocation will be hidden from the patients, evaluators, and statistical analysts. Both groups will manipulate needles in the same way. Unblinding will only be performed in cases of an emergent event.

### Data collection and management

#### Plans for the assessment and collection of outcomes

The data will be collected using CRF, and questionnaires will be implemented by evaluators. The ultrasonographic image-acquisition tests will be performed by the ultrasound physicians. All data obtained during this study will be inputted into the database, anonymized, and stored in the study folder. Only the research team will have access to the particular research folder for this study.

#### Plans to promote participant retention and complete follow-up

The patients will receive sufficient information regarding the study and requirements during recruitment. All patients will be reminded throughout the study to fill in the questionnaires during their study visits. The researchers will continue to collect data and contact patients throughout the follow-up period to ascertain data completeness. Patients will be allowed to discontinue the study, with the data collected up to the withdrawal date anonymized and applied.

#### Data management

The clinical trial management platform ResMan will be used to manage the data, and repeated input methods will be used to ensure that the entered data are correct. The database will be locked with a password that will only be known to the relevant personnel.

#### Confidentiality

Participant names will be abbreviated, and all original medical records and study data will be kept strictly confidential. Only once the trial is completed will the principal investigator submit the data to the third parties. The staff in this study will protect the privacy of participants’ personal medical information as required by law.

### Statistical methods

#### Statistical methods for primary and secondary outcomes

All statistical analyses will be executed with SPSS 22.0 software (IBM SPSS Statistics; IBM Corp, Somers, NY) using the intention-to-treat principle. The CI will be established at 95%, and the significance level at 0.05. For continuous data, the data will be presented as mean ± standard deviation when normally distributed or presented as median (IQR) when not normally distributed. Statistical comparisons will be performed using independent-sample Student’s *t* tests or the Wilcoxon rank-sum test for continuous data, and by ${\chi }^{2}$-test or Fisher exact-probability test for categorical data, as appropriate. A p-value < 0.05 will be considered statistically significant.

#### Methods in our analysis to handle protocol non-adherence and any statistical methods to handle missing data

We will perform statistical analysis on complete cases. The researcher will contact participants as often as possible to supplement missing data, and missing data will be assessed using an intention-to-treat analysis.

#### Plans to give access to the full protocol, participant-level data, and statistical codes

The data sharing will be made available upon reasonable request and with permission and approval from the Beijing Traditional Chinese Medicine Hospital Pinggu Hospital.

#### Oversight and monitoring

To control the quality of this clinical trial, the study’s principal investigator and co-investigator will be responsible for the coordination and data management of each monitoring center. Any decisions that require modification of the study will be made with the consensus of the entire study team after approval by the Ethics Committee of Beijing Traditional Chinese Medicine Hospital Pinggu Hospital. There is no data-monitoring committee for this study.

#### Adverse-event reporting and harm

All adverse events will be documented in the CRF throughout the trial. Adverse events related to acupuncture (such as severe pain, local hematoma, infection and abscess, and broken needles)—including discomfort after treatment—will be recorded in detail in a timely fashion. A detailed description of the categories and severity of adverse events, as well as any correlation with treatment, will be collected by the patients themselves and their evaluators. If the adverse event is severe and associated with the trial, the patient will be withdrawn from the study and provided appropriate medical care.

#### Frequency and plans for auditing trial conduct

The team will be audited every three months, with the Beijing Traditional Chinese Medicine Hospital Pinggu Hospital Trial Steering Committee supervising the trial. The Beijing Clinical Research Quality Promotion Center will also conduct an annual visit to assess the existence and integrity of the investigational documents.

#### Plans for communicating important protocol amendments to the relevant parties (e.g., trial participants and ethics committees)

Any protocol amendments will be reported to the Ethics Committee of the Beijing Traditional Chinese Medicine Hospital Pinggu Hospital, and online trial registries will be updated accordingly.

#### Dissemination plans

The results of this research study will be completely disclosed in international peer-reviewed journals, with both positive and negative results reported.

## Discussion

We aim to evaluate the effectiveness and safety of acupuncture in the treatment of CM compared with sham acupuncture. In recent years, non-penetrating SA has been increasingly advocated as a sham control in acupuncture research [27, 28]. In this study, non-penetrating sham acupuncture will be applied at 14 non-acupuncture points that are not located in the same segments of the breast pain area to evaluate the effect of real acupuncture more accurately.

The assesment of pain is a core outcome in breast pain [29] and the daily VAS-BP has been agreed upon as the recommended for assessing pain [30], it has been suggested that pain ≥ 3 on a VAS of 0 to 10 [31]. The change in the average daily VAS-BP during the first two weeks of menstruation from baseline to endpoints will be the primary outcome. Women experiencing breast pain usually complain its negative impact on their daily life, especially as it pertains to interference with sexual and physical activities, and that it exerts a negative impact on work and social activities. Nevertheless, its effect on QOL is often underestimated [32]. Moreover, the changes in NDBP, WHOQOL-BREF, and global patient assessment will also be assessed in this trial. Some studies on ultrasonograms found that breast structural changes were related to breast pain, and the breast glandular section was thickened (augmented by 7.3%) in the luteal phase compared to the late follicular phase [33]. Thus, breast glandular-section thickness and breast-duct width will be assessed in this trial. As 60% of CM recurs after treatment [4], we will also assess VAS-BP and NDBP 12 and 24 weeks after treatment. We believe that the results of the trial will provide certain evidence of the effects of acupuncture on patients with CM.

Strengths of the study include its multicenter approach (conducted at a tertiary A hospital and two Community Health Service Centers in China), strictly standardized endpoints and objective criteria, long-term follow-up, and strict quality control. The trial also shows some limitations. First, reviewers and statisticians were blinded to reduce bias, but the acupuncturist were not blinded due to the nature of the study, and this could introduce bias into the results. Second, considering ethical norms and acceptance by the participants, we did not assign a waitlist group, which would not preclude the possible spontaneous remission of the CM. Third, we only designed a fixed acupuncture regimen in the trial, which may fail to fully show the efficacy of acupuncture. Therefore, it should be evaluated whether patients benefit from using different acupuncture manipulations (such as twirling, lifting, and thrusting), different treatment time or different acupoints in future studies.

### Trial status

Recruitment began on July 1, 2022, and is anticipated to end on December 31, 2023.

## Data Availability

The individual participant data during the study are available from the corresponding author upon reasonable request.

## Abbreviations

CM: Cyclic Mastalgia
MGH: Mammary Gland Hyperplasia
VAS-BP: Breast Pain VAS
NDBP: Nominal Days of Breast Pain
WHOQOL-BREF: World Health Organization Quality-of-Life Scale-Short Form
QOL: Quality of Life
MA: Manual Acupuncture
SA: Sham Acupuncture
CRF: Case Report Form
